# Morphometric analysis of hippocampus and intracranial formations based on their stages in patients diagnosed with major cognitive disorder

**DOI:** 10.55730/1300-0144.5353

**Published:** 2021-12-15

**Authors:** Nurullah YÜCEL, Muzaffer ŞEKER, Mehmet Tuğrul YILMAZ, Zakir SAKÇI, Yaşar BÜKTE

**Affiliations:** 1 Departmant of Anatomy, Hamidiye Medical Faculty, University of Health Sciences, İstanbul, Turkey; 2 Department of Anatomy, Meram Medical Faculty, Necmettin Erbakan University, Konya, Turkey; 3 Department of Radiology, İstanbul Ümraniye Training and Research Hospital, University of Health Sciences, İstanbul, Turkey

**Keywords:** Alzheimer’s disease, dementia, hippocampus, white matter, volumetric analysis

## Abstract

**Background:**

Alzheimer’s disease (AD) is a major cognitive disorder classified as a common type of dementia. Magnetic resonance imaging (MRI) is the most practical method for diagnostic purposes in AD. The aim of the study was to determine the volume of the hippocampus and intracranial structures in AD using MRI.

**Methods:**

A total of 102 patients with AD were classified based on the mini mental test scores as early, moderate, and advanced stage. The control group included 35 healthy subjects. MRI were compared between the patients and control groups based on the calculations made utilizing *volBrain* software. Intracranial volumetric parameters were also compared between the three stages of AD.

**Results:**

The white matter volumes, total hippocampus, total cerebrum, right cerebrum, left cerebrum, truncus encephalic, total nucleus caudatus and total corpus amygdaloideum were significantly increased in the AD. The white matter volumes, right hippocampus, left hippocampus, total cerebrum, left cerebrum, and right cerebellum were significantly increased in the patients in the early stage compared to the patients in the advanced stage AD.

**Conclusion:**

The most efficient volumetric study in AD could be performed by obtaining long-term periodic morphometric data of an early diagnosed and regularly followed-up patient population.

## 1. Introduction

Alzheimer’s disease (AD) is a progressive neurodegenerative disease accounting for 60%–80% of dementia diseases. Although the cause has not been determined exactly, age is the most important risk factor in the development of AD. The incidence of AD is 0.4% in individuals over 65 years old and 7.6% in those aged over 85 years. New AD cases are reported by 0.4% in people aged under 75 years, 3.2% in those aged between 75–84 years and 7.6% in individuals over 85 years old annually [1]. The global incidence of AD is estimated to exceed 50% in individuals aged over 65 years by 2050 [2]. Therefore, AD is expected to be among the most hazardous health problems in the forthcoming years.

The diagnosis of AD is established according to the criteria of the National Institute of Neurological and Communicative Disorders and Stroke and AD and Related Disorders Association (NINCDS-ADRDA) and The Diagnostic and Statistical Manual of Mental Disorders (DSM) handbook [3]. In order to diagnose and follow-up AD, it is necessary to have both clinical knowledge and the characteristics of the disease course [4].

Numerous imaging methods are used for diagnostic purposes regarding AD today, although magnetic resonance imaging (MRI) is the most practical and easy to access method for this purpose. In addition, it is also possible to diagnose and treat neurodegenerative diseases using MRI [5,6].

According to histological studies, the hippocampus is susceptible to AD disease pathology and is severely damaged when clinical symptoms first appear [7]. Therefore, the hippocampus is the primary target of MRI studies in AD. In parallel to histological findings, longitudinal MRI studies have found increased rates of hippocampus volume loss compared to normal aging [8,9] and mild cognitive impairment [10] in AD compared to normal aging.

Many studies have mentioned temporal and spatial changes in the white matter occurring in the course AD [11,12]. An abnormal white matter volume is associated with poor cognitive performance in AD independently of the cortical gray matter volume [12]. On the other hand, cerebral degeneration studies on patients with AD have shown pathological features in the cortical gray matter [13]. Therefore, cognitive dysfunction may be experienced in patients with AD as a result of changes in white or gray matter.

The objective of this study was to determine hippocampus volumes, substantia alba hyperintensities the volumes of other intracranial structures in AD. For this purpose, these structures were compared between the patients with three stages of AD and healthy subjects using *volBrain* (Manjón ve Coupé 2006) software.

## 2. Material and method

At first, the study protocol was approved by the Necmettin Erbakan University Meram Medical Faculty Ethics Committee for Research Outside Drugs and Medical Devices with the decision (04-27-2018; 2018/1325). The necessary permission to conduct the study was received from the University of Health Sciences, Ümraniye Training and Research Hospital Management.

### 2.1. Patients with AD

Demographic features and MRI data of patients presenting to the neurology outpatient clinic of our hospital with the complaint of forgetfulness, aged over 50 years, who were at least primary school graduate and diagnosed with AD by a neurologist according to the DSM-V (Diagnostic and Statistical Manual of Mental disorders, 5th edition) diagnostic criteria between 1^st^ January 2017 and 31^st^ December 2018 were retrospectively evaluated. Patients’ age, gender, mini-mental test score, diseases, drugs used, and MRI images were obtained from the patient files.

Data of patients with AD (n = 237) were screened. Mini mental test date and cranial MRI date 74 patients who were not compatible mini mental test date and cranial MRI date, 22 patients who were not compatible with mini mentally test scores and clinical findings, 39 patients who were incompatible with exclusion criteria were removed from the study. Finally, patients with AD (n = 102) were included in the study. The patients were classified based on the mini mental test scores as early-stage (31 patients), moderate stage (41 patients), and advanced stage (30 patients).

A total of 35 patients who presented to the neurology outpatient clinic with the complaint of nonspecific headache, who had no complaints of forgetfulness and were not diagnosed with AD based on the DSM-V diagnostic criteria, and who were similar to the patient group in age and gender were included as the control group.

The exclusion criteria included patients under 50 years old, illiterate patients, those with space-occupying lesions in the brain, patients with cerebrovascular diseases, degenerative diseases such as Parkinson diseases, Amyotrophic lateral sclerosis, and essential tremor, and those with primary or metastatic cerebral cancer.

### 2.2. MRI examination of patients

MRI examinations were performed in the radiology clinic of our hospital using 1.5 Tesla GE optima (Waukesha USA) head coil MRI device. T1 weighted MRBravo sequence was set as axial, repetition time (TR) = 1800 ms, echo time (TE) = 3.18 ms, FOV = 200 mm^2^, matrix: 224 × 224 and slice thickness = 1mm. We evaluated the data of our study with MRI for T1 weighted images according to *volBrain* (http://volbrain.upv.es) online volumetric measurement technique as an open-source.

### 2.3. volBrain programming

**Figure 1:**
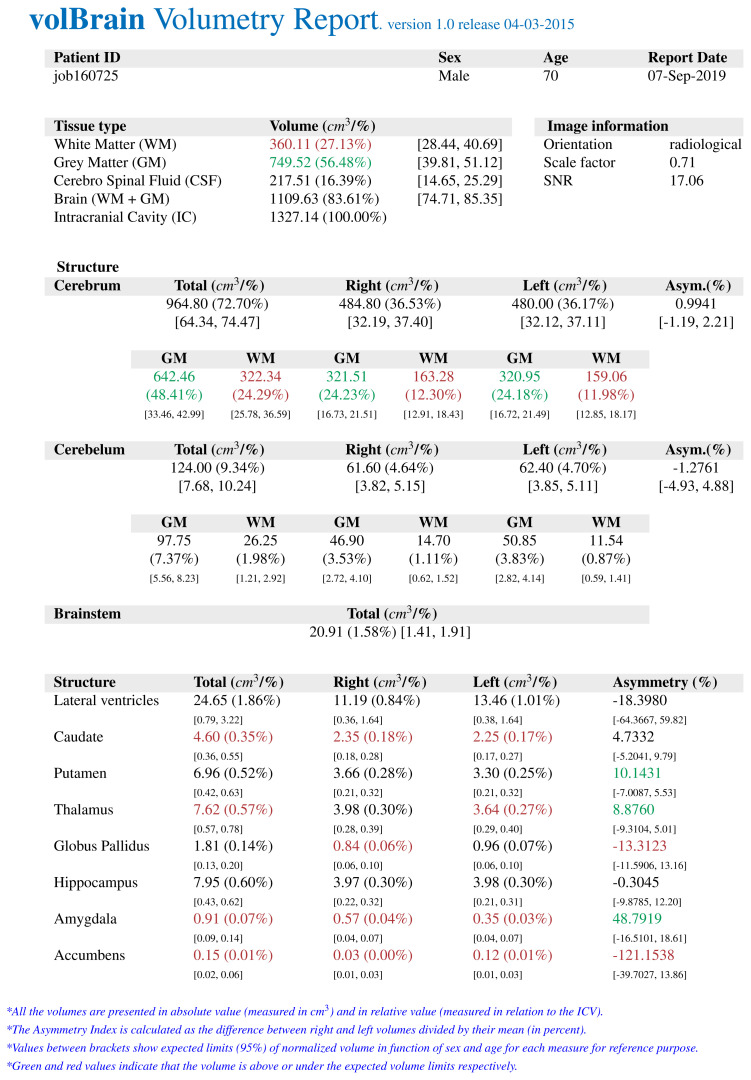
The data obtained from an early-stage AD by *volBrain* volumetry report.

**Figure 2:**
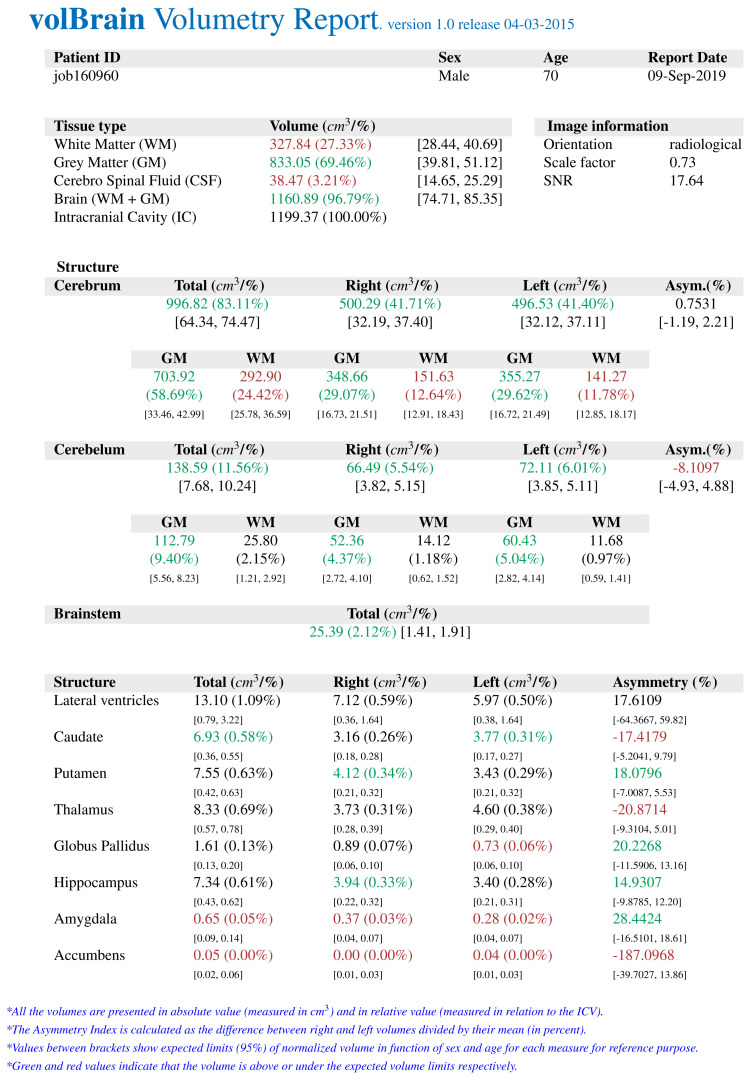
The data obtained from a mild stage AD by *volBrain* volumetry report.

**Figure 3:**
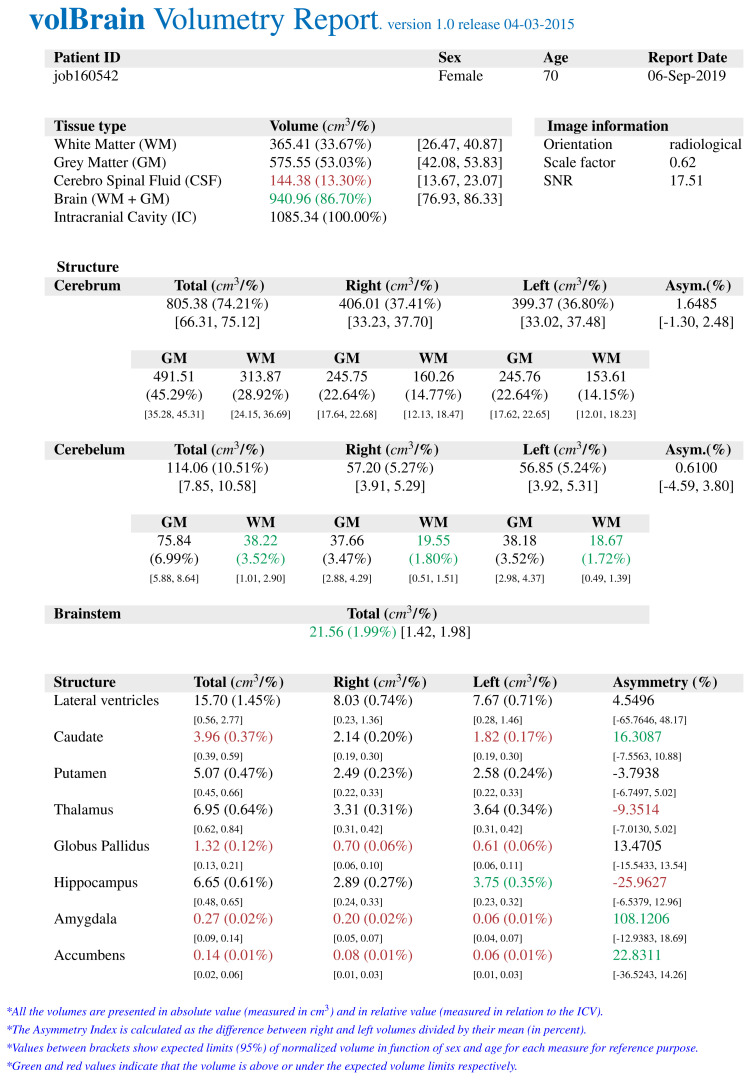
The data obtained from an advanced stage AD by *volBrain* volumetry report.

**Figure 4:**
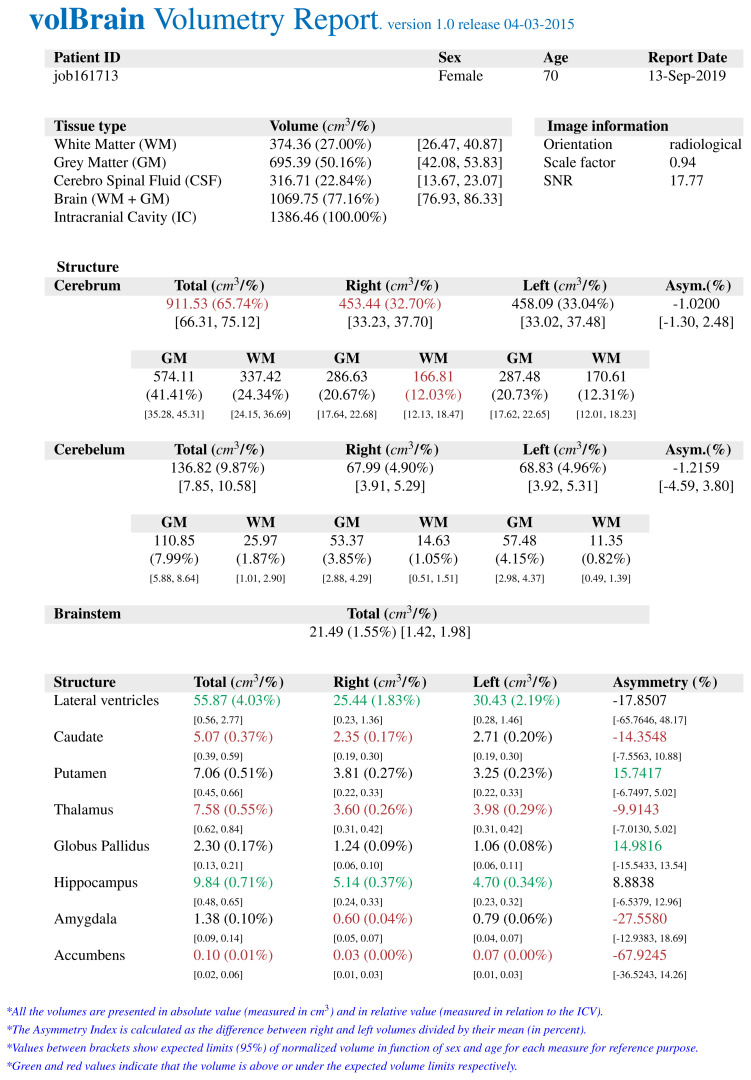
The data obtained from a patient who is in the control group by *volBrain* volumetry report.

The *volBrain* program works fully automatically and allows obtaining volumes of intracranial structures without human interaction. *volBrain* provides volumetric results in a practical, easy, and fast way. It was found by Manjón and Coupé that volumes of the globus pallidus, putamen, and nucleus caudatus were measured manually and with various automatic methods, and *volBrain* method has the highest correlation with the manual method which is currently accepted as the gold standard [14]. There are studies that have shown a high correlation between *volBrain* and manual method which is measured basal ganglia volumes in the literature to compare similar studies [15]. A sample volumetry report of an early-stage AD patient obtained from the *volBrain* is shown in Figure 1. Figure 2, Figure 3, and Figure 4 show volumetric report samples of the moderate stage, advanced stage, and control patients, respectively.

### 2.4. Mini mental state examination (MMSE) test

Mini mental state examination (MMSE) test was developed by Folstein and colleagues in 1975 for dementia screening and is still the most used test today [16]. Total MMSE score consists of 30 points with 10 points measuring orientation to time and place, 3 points registration, 3 points recall, 5 points attention, 8 points language and 1-point visuospatial functions. An MMSE score between 25–30 points is evaluated as normal and a score <25 as a cognitive disorder. MMSE scores between 20–24 points indicate early stage or mild AD, 10–19 points moderate stage AD, and 0–9 points advanced stage AD.

### 2.5. Statistical analysis

Data obtained from the study were statistically analysed using SPSS version 23.0 (Statistical Package for Social Sciences, SPSS Inc., Chicago, IL, USA). In the data analysis, demographic features and parameters of the patients were expressed as descriptive statistics. Comparison between the groups was made with t-test and ANOVA, and LSD test among the post-hoc tests. The relationship of the parameters with age and MMSE scores was evaluated with Pearson’s correlation analysis. The results were evaluated at a 95% confidence interval, p < 0.05 values were considered statistically significant.

## 3. Results

A total of patients with AD (n = 102) were included in the study with being at the early stage (n = 31), moderate stage (n = 41), and advanced stages (n = 30). The control group consisted of patients without AD (n = 35), 51.6% of the patients at the first stage, 58.5% of the patients at the moderate stage, 48.6% of the patients at the advanced stage, and 48.6% of the patients in the control group were females. No statistically significant difference was observed between the groups in terms of gender (p > 0.05). The mean age was significantly increased in the patients at the advanced stage (81.43 ± 7.58) compared to those at the early stage (73.00 ± 6.80), moderate stage (76.83 ± 8.53), and the control group (74.03 ± 4.59) (for all p < 0.001). A comparison of the cerebral volumes between AD patients and the control group is given in Table 1.

The mean values were significantly increased in the control group compared to the AD group in terms of all parameters that showed statistical significance (for all p < 0.05). A comparison of the volumetric values between AD stages is shown in Table 2.

The mean WhiMat, HipoR, HipoL, CerTWM, CerRWM, CerLWM, and CreblR values were significantly increased in the early stage compared to the advanced stage (for all p < 0.05). Whereas, the mean IC, GreyMat, HipoT, Bey, CerT, CerTGM, CerR, CerRGM, CerL, CerLGM, and CreblR parameters were significantly increased in the early stage compared to both the moderate and advanced stages (for all p < 0.05).

According to the AD stages, MMSE results, it was found that hippocampus volumes of the patients at the same stage according to MMSE scores were different.

Patients with AD (n = 103) included in our study, hippocampus total volumes of a male patient (76-year-old) and a female patient (73-year-old) with the lowest MMSE score (4 points) had similar hippocampus total volumes.

However, the mean WhiMat/HipoL, CerTWM/HipoL, and CerLWM//HipoL volume ratios were significantly increased in the AD group than in the control group as hippocampus volumetric rates were compared between the patients with AD and control group (for all p < 0.05).

According to stages of AD, the parameters studied were compared, LatVentT/Hipot ratio was significantly increased in the advanced stage group compared to the patients at the early and moderate stages (p < 0.05). Again LatVentT/HipoT ratio was significantly increased in all stages of AD compared to the control group (p < 0.05).

According to comparison between genders, IC, GreyMat, HipoR, SSS, Bey, CerT, CerTGM, CerR, CerRGM, CerL, CerLGM, CreblR, Mesen, GlobPalT, IC/ThalT, and CerTGM/HipoT parameters were significantly higher in male than in female patients (p < 0.05).

The volumetric parameters of the patients at the early, moderate, and advanced stages were compared according to genders, no significant difference was found between both sexes in terms of volume rates in the patients at the early stage. In the moderate stage; the mean IC, GreyMat, SSS, Bey, CerT, CerTGM, CerR, CerRGM, CerL, CerLGM, CreblR, Mesen, GlobPalT, IC/HipoT, IC/HipoL, IC/CaudT, CerT/HipoT, CerTGM/HipoT, CerTGM/HipoL, CerL/HipoL, and CerLGM/HipoL parameters were significantly increased in the male patients compared to the female patients (for all p < 0.05). Again, in the advanced stage, SSS, IC/CerTWM, and IC/CerLWM parameters were significantly increased in the male than in the female patients (p < 0.05).

There was a negative and weak correlation between GreyMat, HipoT, HipoR, Bey, CerT, CerTGM, CerR, CerL, CerLGM, ThalT, and AmygdT volumes and age (p < 0.05; 0.33 < r <0.00); a negative moderate correlation between CerRGM volume and age (p < 0.05; r = −0.35); and a positive weak correlation between SSS and LatVentT volumes and age (p < 0.05; r_1_ = 0.20, r_2_ = 0.22).

A positive weak correlation was found between IC, WhiMat, GreyMat, HipoT, HipoR, HipoL, Bey, CerTGM, CerTWM, CerRWM, CerLGM, CerLWM, CreblR, Mesen, LatVentT, ThaltT, and CaudT volumes and MMSE (p < 0.05; 0.00 < r < 0.33) while there was a positive moderate correlation between CerT, CerR, CerRGM, and CerL volumes and MMSE (p < 0.05; r_1_= 0.38, r_2_ = 0.37, r_3_ = 0.34, r_4_ = 0.38), and a negative weak correlation between LatVentT volume and MMSE (p < 0.05; r = −0.22).

Lastly, there was a negative moderate between AmygdT volume and age in the patients at the early stage when the correlation between age and MMSE was examined according to the AD stages (p < 0.01; r = −0.393). No significant correlations were found between the volumes, age, and MMSE in the patients at the moderate and advanced AD stages (both p > 0.05).

## 4. Discussion

More than 35 million people have been diagnosed with AD worldwide and this number is expected to double in the next 20 years [17]. AD is a type of dementia, which is a progressive neurological cerebral disease. AD gradually damages the brain by leading to memory loss, language and behavioral problems, and difficulty in performing basic daily tasks [18]. In the brain of a patient with AD, the cortex and hippocampus shrink, damaging the regions involved in thinking, planning, and recall.

Although AD is a gradual disease without known treatment, early diagnosis is essential. Medical and neurological examinations involve separate semistructured interviews with the patient and people who know the patient. In addition, among the imaging methods structural MRI measurements provide a large amount of information in detecting and monitoring the evolution of brain atrophy, which is considered an indicator of AD development. Numerous researchers have used MRI to observe neuronal changes underlying clinical findings of AD. These studies have reported significant volumetric differences in the neocortex and hippocampus of AD patients compared to healthy control subjects [19].

The accuracy of MRI in the diagnosis of AD is 87%. It is possible to measure amygdala, parahippocampus, and hippocampus volumes with MRI volumetric analysis. T1 weighted images are primarily used in the imaging of the hippocampus [20]. The segmentation from MRI sections of the hippocampus can be obtained both manually and automatically [21]. The manual method is a limiting factor in clinical practice as it is both time-consuming and can vary from person to person [14].

In order to solve this problem, automatic multiple atlas identification software such as *volBrain* (http://volbrain.upv.es) is used [14]. Also in our study, we compared hippocampus volumes and substantia alba hyperintensities calculated from the MRI images of the patients at three stages of AD and healthy control subjects utilizing *volBrain* software.

AD is known to be more common among women than in men. In the Baltimore Longitudinal Study, it was found that AD incidence rates in women tended to be higher than men [22] (1.43%/year vs. 1.12%/year). The generally accepted women to men ratio is 2/1 [23]. In our study, women were in the majority among all patients with a rate of 59.8%. These results indicate that our higher rate of women diagnosed with AD is consistent with the studies in the literature.

Although the risks of developing AD are multifactorial, the most important risk factor is aging [24]. The incidence of AD is directly related to age, and the incidence doubles every 5 years after 65 years old. It has been estimated that there were about 5.3 million AD patients in 2015 with 5.1 million being ≥65 years old and 200,000 people under 65 years old who had Early Onset AD (EOAD) [25,26]. In parallel with the literature, in our study, the mean age was significantly increased in the patients at the advanced stage compared to the patients at the other stages and the control group.

In our study we compared *volBrain* measurement results between the AD patients and control group. Accordingly, the mean WhiMat, HipoT, HipoR, CerT, CerTWM, CerR, CerRWM, CerL, CerLWM, Mesen, CaudT, and AmygdT values were significantly increased in the control group compared to the patients with AD (for all p < 0.05). In the current study, volumetric values were also compared between the AD stages. The mean WhiMat, HipoR, HipoL, CerTWM, CerRWM, CerLWM, and CreblR values were significantly higher in the early stage compared to the advanced stage. Whereas, the mean IC, GreyMat, HipoT, Bey, CerT, CerTGM, CerR, CerRGM, CerL, CerLGM, and CreblR parameters were significantly higher in the early stage compared to both the moderate and advanced stages.

In a study by Laakso et al. (2000), changes in hippocampus volumes over three years were evaluated in patients with AD (n = 27) and healthy individuals (n = 8). In that study, the decrease in the hippocampus volume was between 2.2% and 5.8%, in the control group and between 2.3% and 15.6% in the AD patients. However, no significant difference was found between the groups in terms of the decreased rate of hippocampus volumes [27].

Using multiple regional cortical and subcortical volumetric measurements produced by Freesurfer (51 in total), the main purpose of this study was to elucidate the results of these conformation approaches. MRI data were analyzed from two large cohorts, the population-based cohort (N = 406, all subjects 75 years old) and the AD Neuroimaging Initiative cohort (N = 724). The ability of the raw and adjusted hippocampal volumes to predict diagnostic status was also evaluated. In both cohorts, raw volumes correlated positively with intracranial volume. The direction of correlation was reversed for all volume intracranial fractions except lateral and third ventricles. When comparing the estimation of the diagnostic state using different approaches, small but important differences were found. The choice of the normalization approach should be carefully considered when designing a volumetric neuroimaging study [28].

In order to compare hippocampus volumes in different dementia types, Vijayakumar (2012) evaluated MRI images of patients with AD (n = 11), vascular dementia (n = 10), mixed dementia (n = 3), normal pressure hydrocephalus (n = 2), and healthy volunteers (n = 15) using FLD3 procedure. The cognitive functions of the participants were evaluated with MMSE. Hippocampus volumes were found to be shrunk by 25% in AD, 21% in the mixed dementia group, 11% in the vascular dementia group, and 5% in the normal pressure hydrocephalus group. According to the results of that study, hippocampus volume decreases as the severity of dementia increases [29]. In another study by Gerischer et al. (2018), MRI images of AD patients (n = 21) and healthy individuals (n = 21) were evaluated and hippocampus volumes and viscosities of AD patients were found to be lower than healthy individuals [30].

Coupe et al. (2019) evaluated age-related volume changes in the brain in AD. Substantia alba, grey matter, ventriculus lateralis, nucleus caudatus, nucleus accumbens septi, corpus amygdaloideum, hippocampus, putamen, globus pallidus, and thalamus volumes of 3262 AD patients and 2944 healthy volunteers were evaluated using *volBrain* software. According to the study, the reduction in the hippocampus volumes of the AD group started 40 years before healthy volunteers, and the rate of differentiation in ventriculus lateralis and amygdala volumes followed the hippocampus [31].

In particular, the increase in the quality of imaging methods in the recent period and developments in information technologies and morphometric analysis methods enable the provision of morphometric data with more reliable and anatomical borders. With the data obtained from reliable volumetric studies, it has become possible to determine the interaction areas of intracranial anatomical structures according to diseases and to interpret the proportional results.

A second-order activation function is required in individuals at risk for AD. Therefore, the existence of an inverted U-shaped activation pattern is supported [32] and suggests that hyperactivation may represent a biomarker of early AD stages. Accordingly, quantitative brain MRI volumes contribute to the diagnostic identification of behavioral variant frontotemporal dementia from early-onset AD [33]. Percentiles from an MR-based volumetric quantification software program can identify behavioral variant frontotemporal dementia from EOAD. Hippocampal subfield volumes may also play a key role in the diagnostic distinction. Also, large-scale plasma proteomic profiling describes a high-performance biomarker panel for AD screening and staging. This study comprehensively profiled the AD plasma proteome [34]. It is said to serve as a basis for a high-performance, blood-based test for clinical AD screening and staging.

As a limitation, the number of patients should be increased. It is necessary to target specific molecules that play a role in AD, such as acetylcholine, with animal studies following imaging. The new clinical and preclinical results to be obtained will increase the quality of the results in this article. However, we believe that our findings could provide contribution to the literature with using new automatic systems to calculate volumetric values in AD and similar neurodegenerative disorders.

In our study, when “*volBrain* Volumetry Reports” were examined according to MMSE results, it was seen patients who were at the same AD stage based on MMSE score had different hippocampus volumes. We think that since sometimes the MMSE score and the hippocampus volume measurements do not match, the information about the cognitive functions of the patient and the observations and details about the daily life activities obtained from the interviews with the patients and their relatives may be more important in clinical staging.

## 5. Conclusion

We show that the most efficient study can be performed by obtaining long-term periodic morphometric data of an early diagnosed and regularly followed-up patient population.

In future studies on AD and similar neurodegenerative diseases, studying with this methodology will provide healthier data, enabling a more efficient comparison between different studies, contributing to developing diagnostic criteria and treatment performance criteria for AD and similar neurodegenerative diseases. Thus, it could be possible to conduct morphometric analysis studies with a high clinical value.

## Figures and Tables

**Table 1:** Comparison of the cerebral volumes between AD patients and the control group. As defined: Intracranial cavity (IC), White matter (WhiMat), Grey matter (GreyMat), Total hippocampus total (HipoT), Right hippocampus (HipoR), Left hippocampus (HipoL), Central nervous system (SSS), Brain volume (Bey), Total cerebrum (CerT), Grey matter of total cerebrum (CerTGM), White matter of total cerebrum (CerTWM), Right cerebrum (CerR), Grey matter of right cerebrum (CerRGM), White matter of right cerebrum (CerRWM), Left cerebrum (CerL), Grey matter of left cerebrum (CerLGM), White matter of left cerebrum (CerLWM), Total cerebellum (CerblT), Left cerebellum (CerblR), Right cerebellum (CerblL), Truncus encephali (Mesen), Total nucleus caudatus (CaudT), Total putamen (PutamT), Total globus pallidus (GlobPalT), Total corpus amygdaloideum (AmygdT).

	AD	Control	p
IC	1329.36 ± 140.62	1357.28 ± 137.06	0.310
WhiMat	373.80 ± 47.72	423.94 ± 57.50	**0.000**
GreyMat	653.95 ± 73.61	682.18 ± 79.04	0.057
HipoT	7.31 ± 1.05	7.82 ± 1.19	**0.019**
HipoR	3.71± 0.59	3.97 ± 0.63	**0.030**
HipoL	3.66 ± 0.55	3.84 ± 0.64	0.097
SSS	295.69 ± 75.79	383.33 ± 525.08	0.332
Bey	1026.23 ± 138.17	1354.52 ± 1449.05	0.190
CerT	895.24 ±92.05	960.82 ± 96.63	**0.000**
CerTGM	562.14 ± 68.72	587.29 ± 65.46	0.061
CerTWM	333.10 ± 42.99	373.52 ± 49.79	**0.000**
CerR	448.70 ± 47.22	480.71 ± 48.18	**0.001**
CerRGM	280.48 ± 34.64	293.42 ± 31.80	0.054
CerRWM	168.22 ± 22.23	187.29 ± 23.86	**0.000**
CerL	446.54± 45.79	480.11 ± 48.75	**0.000**
CerLGM	281.66 ± 34.57	293.88 ± 33.97	0.072
CerLWM	164.88 ± 21.66	186.23 ± 26.77	**0.000**
CerblT	118.40 ± 16.12	123.85 ± 14.72	0.080
CreblR	59.36 ± 6.22	60.03 ± 11.65	0.667
CreblL	59.62 ± 8.19	62.07 ± 7.72	0.124
Mesen	20.47 ± 2.37	21.50 ± 3.21	**0.046**
LatVentT	34.42 ± 17.08	29.96 ± 18.12	0.192
ThalT	6.90 ± 1.21	7.20 ± 0.86	0.172
CaudT	4.72 ± 1.05	5.34 ± 0.72	**0.001**
PutamT	6.29 ± 1.08	6.41 ± 1.29	0.595
GlobPalT	1.73 ± 0.45	1.79 ± 0.68	0.647
AmygdT	0.77 ± 0.43	0.95 ± 0.36	**0.030**

**Table 2:** Comparison of the volumetric values between AD stages. As defined: Intracranial cavity (IC), White matter (WhiMat), Grey matter (GreyMat), Total hippocampus total (HipoT), Right hippocampus (HipoR), Left hippocampus (HipoL), Central nervous system (SSS), Brain volume (Bey), Total cerebrum total (CerT), Grey matter of total cerebrum (CerTGM), White matter of total cerebrum (CerTWM), Right cerebrum (CerR), Grey matter of right cerebrum (CerRGM), White matter of right cerebrum (CerRWM), Left Cerebrum (CerL), Grey matter of left cerebrum (CerLGM), White matter of left cerebrum (CerLWM), Total cerebellum (CerblT), Left cerebellum (CerblR), Right cerebellum (CerblL), Truncus encephali (Mesen), Total nucleus caudatus (CaudT), Total putamen (PutamT), Total globus pallidus (GlobPalT), Total corpus amygdaloideum (AmygdT).

	Early AD	Moderate AD	Advanced AD	p
IC	1390.02 ± 140.57	1308.76 ± 139.25	1294.85 ± 126.17	**0.013**
WhiMat	392.88 ± 53.83	372.27 ± 43.54	356.18 ± 40.04	**0.009**
GreyMat	687.58 ± 67.94	644.07 ± 58.93	632.72 ± 86.55	**0.007**
HipoT	7.77 ± 1.00	7.18 ± 1.03	7.01 ± 1.02	**0.011**
HipoR	3.92 ± 0.53	3.68 ± 0.59	3.54 ± 0.59	**0.033**
HipoL	3.84 ± 0.58	3.66 ± 0.46	3.46 ± 0.56	**0.020**
SSS	292.78 ± 74.85	290.39 ± 83.63	305.95 ± 66.34	0.676
Bey	1087.56 ± 89.19	1007.17 ± 175.77	988.90 ± 98.32	**0.009**
CerT	943.38 ± 80.61	889.51 ± 88.45	853.33 ± 87.50	**0.000**
CerTGM	595.20 ± 54.39	556.41 ± 66.39	535.81 ± 73.33	**0.002**
CerTWM	348.18 ± 47.55	333.10 ± 41.09	317.53 ± 35.70	**0.019**
CerR	473.04 ± 40.26	446.57 ± 44.20	426.47 ± 47.36	**0.000**
CerRGM	297.21 ± 27.05	278.05 ± 32.53	266.51 ± 38.12	**0.002**
CerRWM	175.83 ± 24.31	168.52 ± 21.60	159.96 ± 18.26	**0.019**
CerL	470.35 ± 41.22	442.94 ± 45.17	426.86 ± 41.30	**0.001**
CerLGM	297.99 ± 27.94	278.36 ± 34.41	269.30 ± 35.55	**0.003**
CerLWM	172.36 ± 23.66	164.59 ± 20.50	157.56 ± 18.96	**0.027**
CerblT	123.14 ± 10.10	116.74 ± 20.67	115.76 ± 13.24	0.141
CreblR	61.39 ± 5.21	59.17 ± 6.08	57.52 ± 6.91	**0.049**
CreblL	61.74 ± 5.28	59.01 ± 10.60	58.24 ± 6.53	0.207
Mesen	21.09 ± 2.38	20.47 ± 2.32	19.84 ± 2.32	0.119
LatVentT	31.57 ± 14.33	31.98 ± 17.76	40.69 ± 17.64	0.055
ThalT	7.26 ± 1.01	6.85 ± 1.35	6.59 ± 1.14	0.088
CaudT	4.86 ± 0.92	4.83 ± 1.00	4.43 ± 1.20	0.188
PutamT	6.32 ± 0.83	6.28 ± 1.21	6.27 ± 1.15	0.985
GlobPalT	1.73 ± 0.38	1.81 ± 0.55	1.64 ± 0.34	0.313
AmygdT	0.80 ± 0.36	0.83 ± 0.43	0.65 ± 0.48	0.211
